# Developmental and Functional Interactions Structure Patterns of Variational Modularity in the Lunar Wrasse Skull

**DOI:** 10.1093/icb/icaf099

**Published:** 2025-06-20

**Authors:** April L Hugi, Howan Chan, Andrea Rummel, Hiroyuki Motomura, Yuna Dewa, Midori Matsuoka, Masayuki C Sato, Olivier Larouche, Kory M Evans

**Affiliations:** Department of Biosciences, Rice University, 77005, Houston, United States; Department of Biosciences, Rice University, 77005, Houston, United States; Department of Biosciences, Rice University, 77005, Houston, United States; University Museum, Kagoshima University, 890-8580, Kagoshima, Japan; United Graduate School of Agricultural Sciences, Kagoshima University, 890-8580, Kagoshima, Japan; Faculty of Fisheries, Kagoshima University, 890-8580, Kagoshima, Japan; United Graduate School of Agricultural Sciences, Kagoshima University, 890-8580, Kagoshima, Japan; Department of Biology, Western Carolina University, 28723, Cullowhee, United States; Department of Biosciences, Rice University, 77005, Houston, United States

## Abstract

Trait modularity is a defining feature of complex life. However, the drivers of modularity across different scales of biological organization remain opaque. Studies have shown that a combination of developmental and functional interactions can structure patterns of trait covariation at the developmental, population, and even macroevolutionary level. However, it remains unclear how developmental and functional interactions may translate or influence macroevolutionary patterns of trait covariance and diversification. Pharyngognathy is a striking evolutionary innovation that has evolved multiple times in acanthomorph fishes and has resulted in the evolution of robust pharyngeal jaws that are used to process hard prey. Recent studies have found strong patterns of evolutionary integration among the jaw systems in pharyngognathous fishes suggesting that this innovation was brought about by the evolutionary coupling of two otherwise distinct trait complexes. Furthermore, the pharyngeal jaws have been hypothesized to act as a constraining force on the evolution of the oral jaws potentially due to their developmental origins in the more conserved *hox-*positive region of the skull. While multiple studies have recovered strong evolutionary integration between the jaw systems, patterns of modularity at the population (variational) level appear to differ, where a high degree of modularity has been found between the oral and pharyngeal jaws suggesting a disconnect between patterns of evolutionary modularity and patterns of variational modularity. Here, we are using three-dimensional geometric morphometrics to test for modularity between the oral and pharyngeal jaws at the variational level in a population of Lunar wrasse collected from Kagoshima, Japan and additionally test for differences in morphological disparity between the oral and pharyngeal jaws. We find strong support for a developmental hypothesis of modularity that separates the jaw systems into distinct modules. We additionally find mixed support for the constraint hypothesis of the pharyngeal jaws, where some elements of the pharyngeal jaws were found to exhibit less morphological disparity than the oral jaws while others exhibited more morphological disparity. Our findings suggest that developmental and functional interactions at the variational level may impart patterns of covariation that are distinct from evolutionary patterns of modularity that are found between species.

## Introduction

Understanding how phenotypic traits covary and identifying the forces that shape these patterns are central goals in evolutionary biology. Developmental, functional, genetic, and environmental forces act on phenotypes and result in unevenly dispersed patterns of covariation across traits, i.e., some subsets of traits will exhibit higher degrees of covariance with one another, while other traits will exhibit less covariance among themselves (Olson and Miller 1958; Wagner 1996; Schlichting and Pigliucci 1998; Klingenberg 2008). Morphological integration refers to covariation among traits, while modularity occurs when this integration is organized into distinct complexes (modules, e.g., jaw, cranium, and limb) with strong internal covariation and weak covariation between groups (Klingenberg 2014). Such modular organization is widespread across biological systems (Klingenberg 2004, 2014; Zelditch and Goswami 2021) and is thought to promote evolutionary flexibility. By allowing modules to evolve somewhat independently, modularity has been hypothesized to function as an adaptive innovation and may facilitate the evolution of complex forms and increase morphological diversity over time (McShea 1996; Schlosser and Wagner 2004; Vermeij 2015). In contrast, strong morphological integration can constrain evolution by channeling trait changes along specific evolutionary trajectories (Klingenberg 2008).

Studies have shown that modular trait complexes co-evolve over evolutionary timescales and that these modular associations often reflect functional interactions between traits but may sometimes also reflect shared developmental origins (Felice and Goswami 2018; Larouche et al. 2018, 2023; Du et al. 2019; Watanabe et al. 2019; Conith and Albertson 2021; Conith et al. 2021; Rhoda et al. 2021a). Interestingly, while patterns of evolutionary modularity and integration have been well-documented in the literature, studies of population-level patterns of trait covariation are far less common and the few representative studies that compare macroevolutionary and population-level patterns of trait covariation have often found discordant results, where patterns of modularity differ between timescales and scales of biological organization (Badyaev et al. 2005; Evans et al. 2023). The lack of understanding of how modularity shapes patterns of trait covariation at the population level represents a large gap. Evolution occurs at the population level, and many organismal traits exhibit plasticity and are shaped in real time by developmental processes, behavior, and functional interactions (Conith et al. 2019; Lofeu et al. 2024). Therefore, understanding population-level patterns of trait covariation can yield valuable insights into how patterns of trait covariation may manifest and ultimately influence macroevolutionary patterns. Population-level patterns of trait covariation may also yield valuable insights about how developmental and functional interactions at the population level may influence the modular configuration of organisms.

Pharyngonathy is a striking evolutionary innovation that consists of the fusion of the fifth ceratobranchial bones into robust tooth plates, a diarthrosis between the dorsal surface of the upper pharyngeal jaw bones and the underside of the neurocranium, and the presence of a muscular sling that connects the underside of the neurocranium with the lower pharyngeal tooth plate (Wainwright et al. 2012). Together this innovation allows fishes to crush duraphagous prey (Liem 1973). This innovation has evolved multiple times independently across several clades of fishes including cichlids and labrids (Mabuchi et al. 2007; Wainwright et al. 2012). When originally proposed as a phylogenetic character, pharyngognathy was hypothesized to function as an evolutionary “key innovation,” which allowed fishes to functionally decouple their oral jaws and pharyngeal jaws: the oral jaws specialize in prey capture, while the pharyngeal jaws specialize in prey processing and therefore act as separate modules. This innovation was hypothesized to be a key driver in the success of the large cichlid and labrid radiations (Liem 1973; Kaufman and Liem 1982; Liem and Sanderson 1986; Wainwright et al. 2012).

Today, there is limited support for much of the pharyngognathy hypothesis. Functional decoupling of the jaws in the case of pharyngonathy does not necessarily lead to expanded function or morphological diversity. A recent study found that pharyngognathy actually appears to limit morphological and functional diversification of feeding structures when compared to lineages without this trait. The modified pharyngeal jaws are more robust and powerful and have increased prey processing capacity but have a restricted pharyngeal jaw gape and are kinetically constrained (Roberts-Hugghis et al 2025). Phylogenetic comparative studies have shown that rates of speciation and morphological evolution are not dependent upon the presence or absence of pharyngognathy (Larouche et al. 2020; Borstein et al. 2024). Additionally, Alfaro et al. (2009) found that while labrid fishes diversified rapidly compared to other percomorph fishes, there is no evidence that this diversification was driven by pharyngeal jaw innovations. There is also uncertainty concerning the evolutionary decoupling between oral and pharyngeal jaws in pharyngognathous fishes (Hulsey 2006; Burress and Muñoz 2021; Roberts-Hugghis et al. 2023). For example, in cichlid jaw systems some studies found support for integrated or coupled jaw systems (Conith and Albertson 2021) while others propose they are modular or decoupled (Hulsey et al. 2006; Burress et al. 2020; Ronco and Salzburger 2021). Previous work on the macroevolutionary modularity patterns of the cranial morphology of labrid fishes showed support for functional modularity hypotheses with strong support for a modularity model that divided bones into a slow evolving feeding module (oral and pharyngeal jaws; coupled jaw system) and breathing module (hyoid apparatus and operculum) (Larouche et al. 2023), which suggest that the oral and pharyngeal jaws co-evolved and may have even constrained each other during evolution. Similarly, Roberts-Hugghis et al (2025) found constrained phenotypic diversity and stronger trait coevolution in pharyngognathous fishes compared to non-pharyngognathous fishes.

The lack of evidence for decoupling at the evolutionary level is perhaps surprising because studies have shown that within an organism, the oral and pharyngeal jaws do function separately and are governed by different sets of muscles with distinct activation patterns (Liem 1973). It has been hypothesized that ancient, shared gene-interaction networks that form the dentition of both systems are responsible for maintaining the integration between the oral and pharyngeal jaws and that these shared interaction networks may result in the co-evolution of the two jaws systems despite functioning separately within an organism (Fraser et al. 2009). However, to date few studies have tested the decoupling hypothesis in phayngognathous fishes at the population level (Hulsey 2006; Chan et al. 2024). Therefore, we lack a robust understanding of the factors that govern patterns of trait covariation within these oral and pharyngeal jaws systems.

In addition to patterns of covariation between the oral and pharyngeal jaws, the differences in the developmental origin between these jaw systems may play a role in structuring downstream patterns of morphological diversification. Studies in mammals have shown that differences in developmental origin can impact rates of trait diversification, where regions derived from cranial neural crest evolve more quickly than bones derived from paraxial mesoderm. (Goswami et al. 2023). Similarly, there is a growing body of literature that suggests that *hox-*derived pharyngeal jaws are more constrained than the oral jaws, which are derived from cranial neural crest and mesoderm (Fraser et al. 2009). In labrid fishes, it was found that the pharyngeal jaws evolve under a single-peak Ornstein–Uhlenbeck model, which suggests stabilizing selection, while several of the oral jaw elements evolve under a Brownian motion model (Larouche et al. 2023). Even at the developmental level in parrotfishes, the lower pharyngeal tooth plate exhibits less allometric growth than the bones of the oral jaws (Neves et al. 2024). In neotropical cichlids, a recent study also found strong support for stabilizing selection across the pharyngeal jaws as opposed to the early-burst model that has been typically recovered in other cichlid macroevolutionary studies (Nicholas and López-Fernández 2024). Other broader studies of pharyngognathous fishes have found that species that exhibit pharyngognathy exhibit less functional diversity than species that lack this innovation (Roberts-Hugghis et al. 2025). These studies not only suggest that the pharyngeal jaws may be under more constraint than the oral jaws, but that through their strong evolutionary integration with the oral jaws, may constrain morphological diversification in the trophic apparatus in general.

Here, we test the decoupling hypothesis at the population level in the lunar wrasse, *Thalassoma lunare*, collected from Kagoshima Bay, Japan, near the base of Sakurajima to understand how pharyngognathy structures patterns of trait covariation at the organismal level. We hypothesize that the two jaw systems will comprise distinct modules at the population level because they function independently and have distinct developmental origins. Additionally, we test for the effect of developmental origin on patterns of morphological disparity among the different cranial modules. We hypothesize that the *hox-*derived pharyngeal jaws will exhibit less morphological disparity than the neural crest and mesodermally derived oral jaws.

## Methods

### Field sampling

**Fig. 1:**
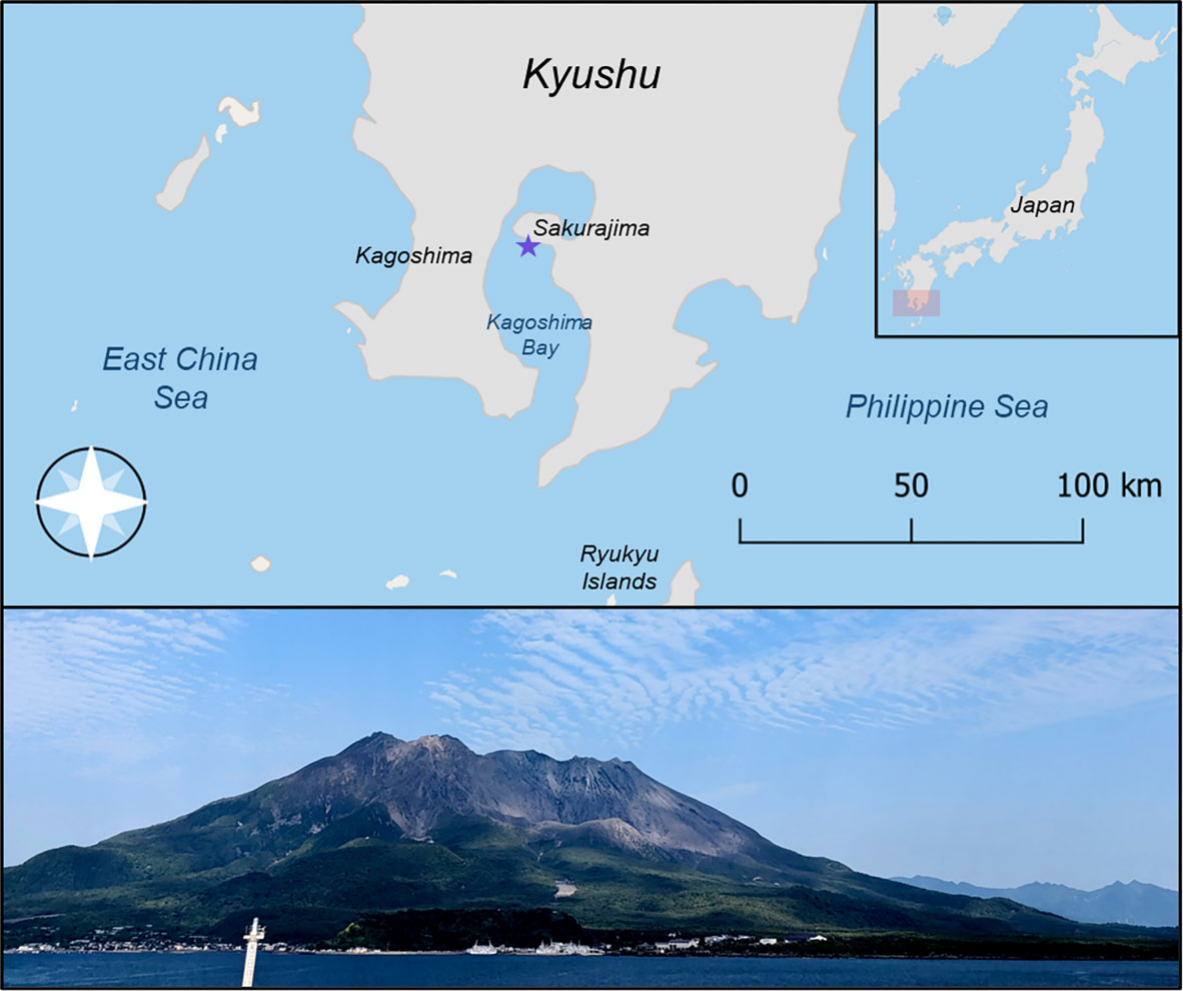
The top panel highlights the study site where lunar wrasse were collected with a star in Kagoshima Bay just south of the stratovolcano, Sakurajima (pictured in the bottom panel).

Fish were collected on May 17th, 2023, just south of Sakurajima volcano in Kagoshima Bay, southern Kyushu, Japan (Fig. 1). The fish were collected following IACUC guidelines by SCUBA divers from Rice University and Kagoshima University. Divers set up stationary nets on the seafloor, where they collectively corralled groups of fish. A total of 36 specimens were collected ranging in size from 16 to 20 cm TL. To reduce the effect of ontogeny on our shape analyses, we limited our sampling to reproductively mature males and females. Fish were preserved in 10% buffered formalin and later transferred to 70% ethanol for long term storage.

### Geometric morphometrics

**Fig. 2:**
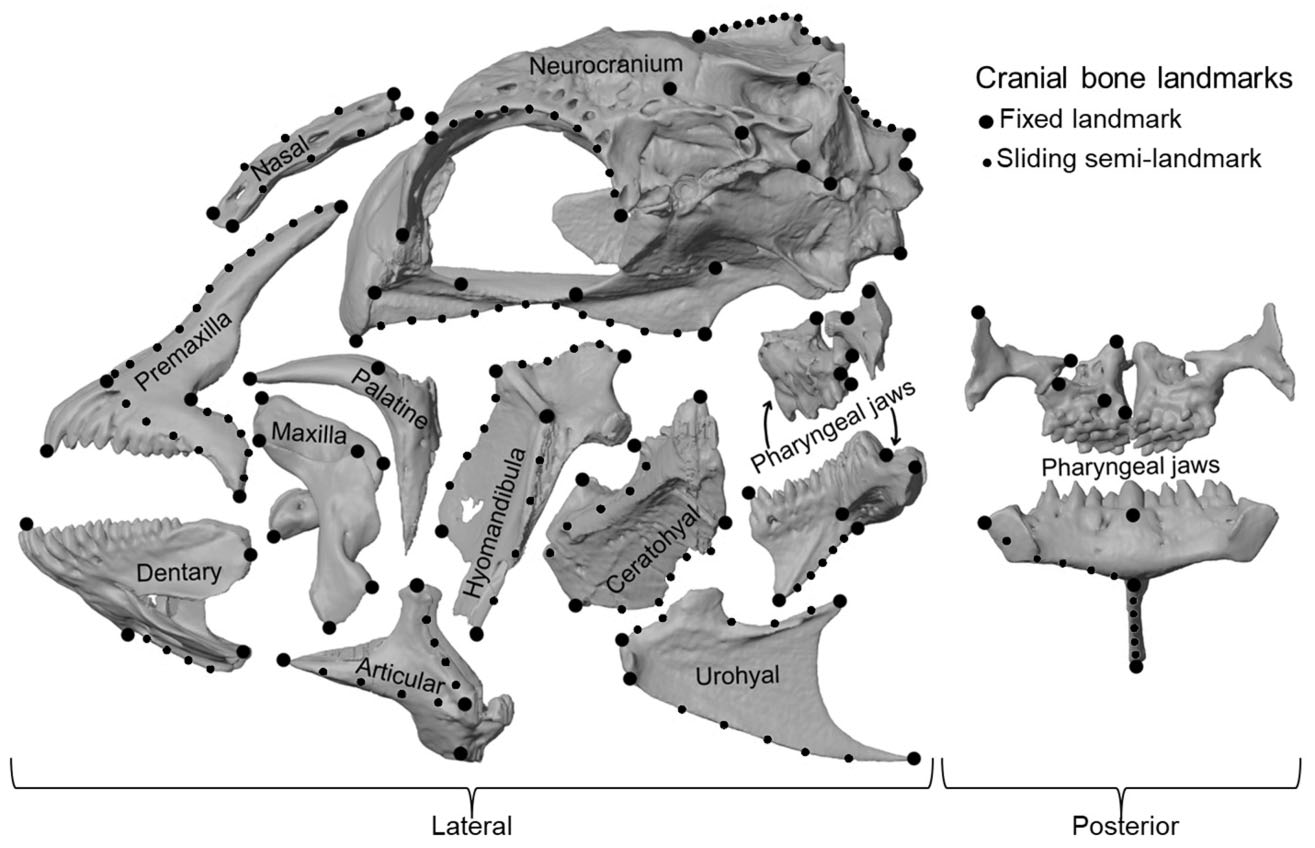
Cranial bone landmark positions excluding the preopercular/opercular series. Articular represents the fused articular and angular bones.

The skull shape of the lunar wrasse was quantified by micro-computed tomography (CT) with a Bruker Skyscan 1273. Scans were shot in high resolution around 35.5 um, 70 kV, and 300 uA. Reconstructions were segmented with Amira software (Thermo Fisher Scientific, Waltham, MA) to isolate the cranial bones, and volumetric data was converted into 3D meshes (isosurfaces). 3D meshes were then landmarked in Stratovan Checkpoint following the landmark scheme in Fig. 2 (Table S1 for complete list) with landmarks defining notable features and margins of bones that represent the oral jaws, pharyngeal jaws, neurocranium, and hyoid regions. Only the left side of the skull reconstruction was digitized to increase statistical power. A generalized Procrustes analysis (GPA) in the R package, GEOMORPH (Adams et al. 2016)., aligned bones and removed non-shape variation (i.e., size and orientation) (Rohlf and Slice 1990). Because wrasse skulls are kinetic articulating structures, a local superimposition was performed to account for any artificial disparity between specimens because of differences in arbitrary resting position during CT scanning (Rhoda et al. 2021a, 2021b). For the local superimposition, the GPA-translated landmarks from each bone were superimposed onto a mean shape derived from the GPA of the entire landmark array of all 36 wrasse specimens.

### Modularity testing

**Fig. 3:**
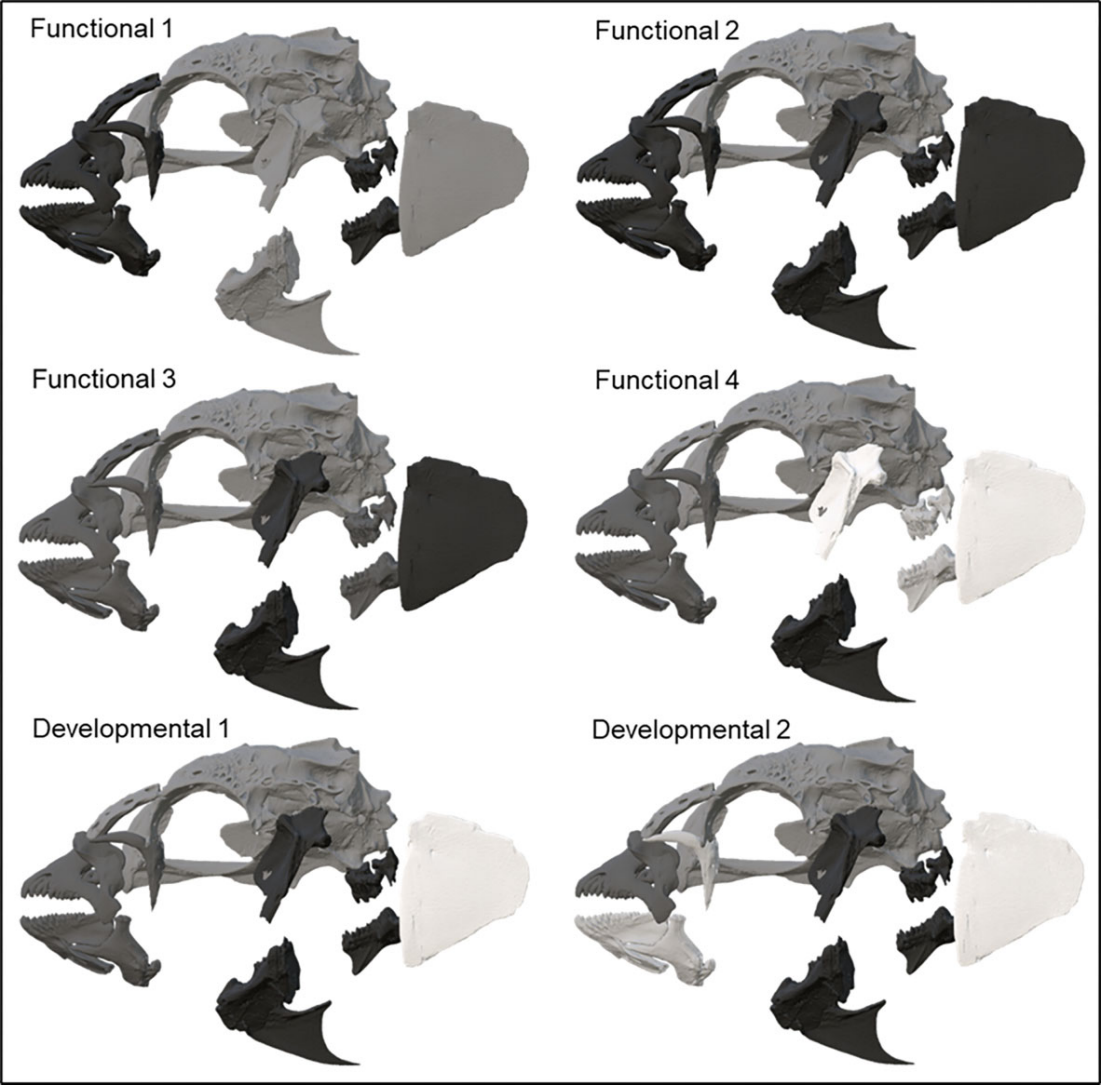
Modularity hypotheses for cranial bones of *Thalassoma lunare*. Each color represents a different module. The operculum represents the preopercular/opercular series. See Table 1 for a description of the functional and developmental patterns that underly each hypothesis.

We evaluated six hypotheses of modularity following a study of evolutionary modularity in the wrasse skull by Larouche et al. (2023). Our six hypothesized modular subdivisions consisted of four functional hypotheses based on the kinetic relationships of the skull bones during feeding, breathing, and more specifically prey capture, and prey processing and two developmental hypotheses based on the developmental origins and interactions primarily related to the pharyngeal arch series and their derivatives starting with the mandibular and hyoid arch derivatives and then subdividing those arches into dorsal and ventral mandibular arch derivatives. All hypotheses were based on literature reviews for relevant biomechanical and developmental biology studies (Table 1, Fig. 3). We also included a total integration (1 module) hypothesis as a null model. We used the covariance ratio (CR) to quantify the ratio of between-module covariance to within-module covariance. CR values greater than zero and less than one describe data sets, where the degree of covariation between modules is less that that found within modules and thus a high degree of modularity (Adams 2016). The functions “modularity.test” and “compare.CR” from the R package, GEOMORPH, were used to calculate CRs and effect sizes, respectively for each modularity hypothesis. To provide additional support for modularity hypotheses, we used graphical modeling with a distance-matrix approach to evaluate conditional independence among shape partitions (Monteiro et al. 2005). Graphical models depict variables as nodes in a graph and the conditional independence of the variables as edges that connect the nodes. Because of the theoretical importance of conditional independence for the presence of modularity, the degree of conditional dependence can be evaluated using an edge exclusion deviance as a deviance information criterion that measures whether a particular edge can be removed from a model that includes it. Different hypotheses of modularity can then be evaluated for their fit to the data using this information criterion (Zelditch and Goswami 2021). To evaluate our hypotheses of modularity, pairwise Procrustes distances were calculated among individuals for each shape partition and used to generate a correlation matrix. We then used graphical modeling to test for conditional independence within and between shape partitions using the “fitConGraph” function from the R package ggm 2.5.1 (Chan et al. 2024; Marchetti et al. 2024). Graphical models were compared with the Akaike information criterion (AIC) (Akaike 1974).

**Table 1:** Modularity hypotheses from Larouche et al. (2023)

Hypothesis	Description of modules	References
*Functional 1*	Feeding + buccal expansion	Liem (1973), Westneat (2004), Hulsey et al. (2005), Camp and Brainerd (2014), Camp et al. (2015)
*Functional 2*	Feeding and buccal expansion + neurocranium	Liem (1973), Westneat (2004), Hulsey et al. (2005), Camp and Brainerd (2014), Camp et al. (2015)
*Functional 3*	Feeding + buccal expansion + neurocranium	Liem (1973), Westneat (2004), Hulsey et al. (2005), Camp and Brainerd (2014), Camp et al. (2015)
*Functional 4*	Prey capture + prey processing + lateral buccal expansion + ventral buccal expansion + neurocranium	Liem (1973), Westneat (2004), Hulsey et al. (2005), Camp and Brainerd (2014), Camp et al. (2015)
*Developmental 1*	Mandibular arch derivatives + hyoid and pharyngeal arch derivatives + neurocranium and nasals + operculum	Hulsey et al. (2005), Le Pabic et al. (2016)
*Developmental 2*	Dorsal mandibular arch derivatives + ventral mandibular arch derivatives + hyoid and pharyngeal arch derivatives + neurocranium and nasals + operculum	Cerny et al. (2004), Hulsey et al. (2005), Le Pabic et al. (2016)

### Integration

Integration of each bone was estimated with the relative eigenvector index (V_rel_), which was calculated with the integration.Vrel’ function in the R package geomorph. V_rel_ measures the overall degree of covariation within each individual bone (Pavlicev et al. 2009; Conaway and Adams 2022). A two-block partial least squares analysis was conducted with the “integration.test” function in the R package geomorph to examine pairwise integration between each individual bone (Rohlf and Corti 2000; Adams and Collyer 2019). The function, ‘integration.test” returns a matrix of PLS correlations for each pair of bones. Significance testing was performed by 1000 permutations. A network plot was created to visualize the within (nodes) and between (edges) bone patterns of integration across the skull.

### Morphological disparity

Traits can differ in their range of variation and the developmental origin of traits can have downstream effects on not only the range of variation produced, but also in the relative rate of evolution among sets of traits (Evans et al. 2017a, 2017b). To test for differences in morphological disparity between the bones of the lunar wrasse skull, we quantified morphological disparity using the “morphol.disp” function in the R package geomorph 4.0.7. To allow comparisons of disparity between bones and modules (groups of bones), disparity estimates were standardized by dividing by the total number of landmarks included in each bone or module. Morphological disparity was quantified for individual cranial bones and for the five modules that make up the modularity hypothesis that received the strongest statistical support in this analysis.

## Results

### Modularity testing

**Fig. 4:**
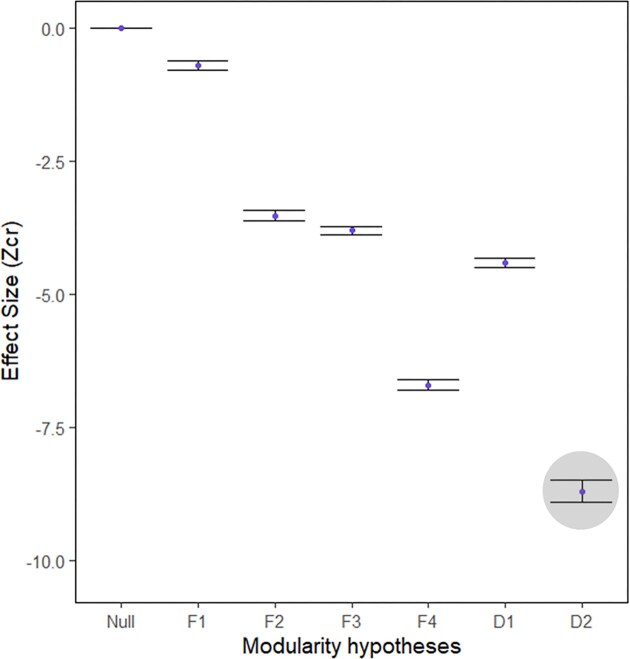
Effect size of CRs for Modularity hypotheses (Table 2, Fig. 2) and their 95% confidence intervals. The best supported hypothesis is highlighted.

The lunar wrasse skull exhibits a high degree of variational modularity. All modularity hypotheses except for Functional 1 had CRs that were significantly lower than the null distribution. There is a linear relationship between the number of modules included in the hypothesis and the effect size. Excluding the “All separate” hypothesis, which treats all 13 bones as independent modules, Developmental 2 has the lowest Z_CR_ and thus is the best hypothesis to describe the modular structure of the skull (Table 2, Fig. 4).

**Table 2:** Modularity testing results for six different hypotheses described in Table 1. Effect size based on 1000 permutations. For CR, lower effect sizes reflect higher modularity. Bold fonts reflect the best-fitting model for CR and graphical modeling

		CR			Graphical modeling	
Hypothesis	Number of modules	CR	*P*-value	Z_CR_	Deviance	AIC
Total integration	1	NA	NA	0	0	0
Functional 1	2	0.968	0.241	−0.697	54.379	−25.621
Functional 2	2	0.581	0.001	−3.523	18.887	−5.113
Functional 3	3	0.718	0.001	−3.800	63.536	−24.464
Functional 4	5	0.635	0.001	−6.707	69.022	−50.978
Developmental 1	4	0.614	0.001	−4.412	50.630	−63.370
Developmental 2	5	0.616	0.001	**–8.702**	51.914	**–74.086**
All separate	13	0.590	0.001	−10.150	84.542	−71.458

Results of the distance-matrix approach (graphical modeling) support the developmental modularity hypotheses, where cranial bones are divided into modules based on the pharyngeal arch series and their derivatives with strongest support for Developmental 2 (Table 2).

### Integration

The oral and pharyngeal jaws are significantly decoupled at the population level in the lunar wrasse. A two-block partial least squares analysis between the oral and pharyngeal jaw modules recovered a non-significant relationship with an rPLS of 0.68, and effect size of 0.145 and a *P*-value of 0.43 based on 1000 random permutations. This finding suggests that the oral and pharyngeal jaws are significantly decoupled from each other.

**Fig. 5:**
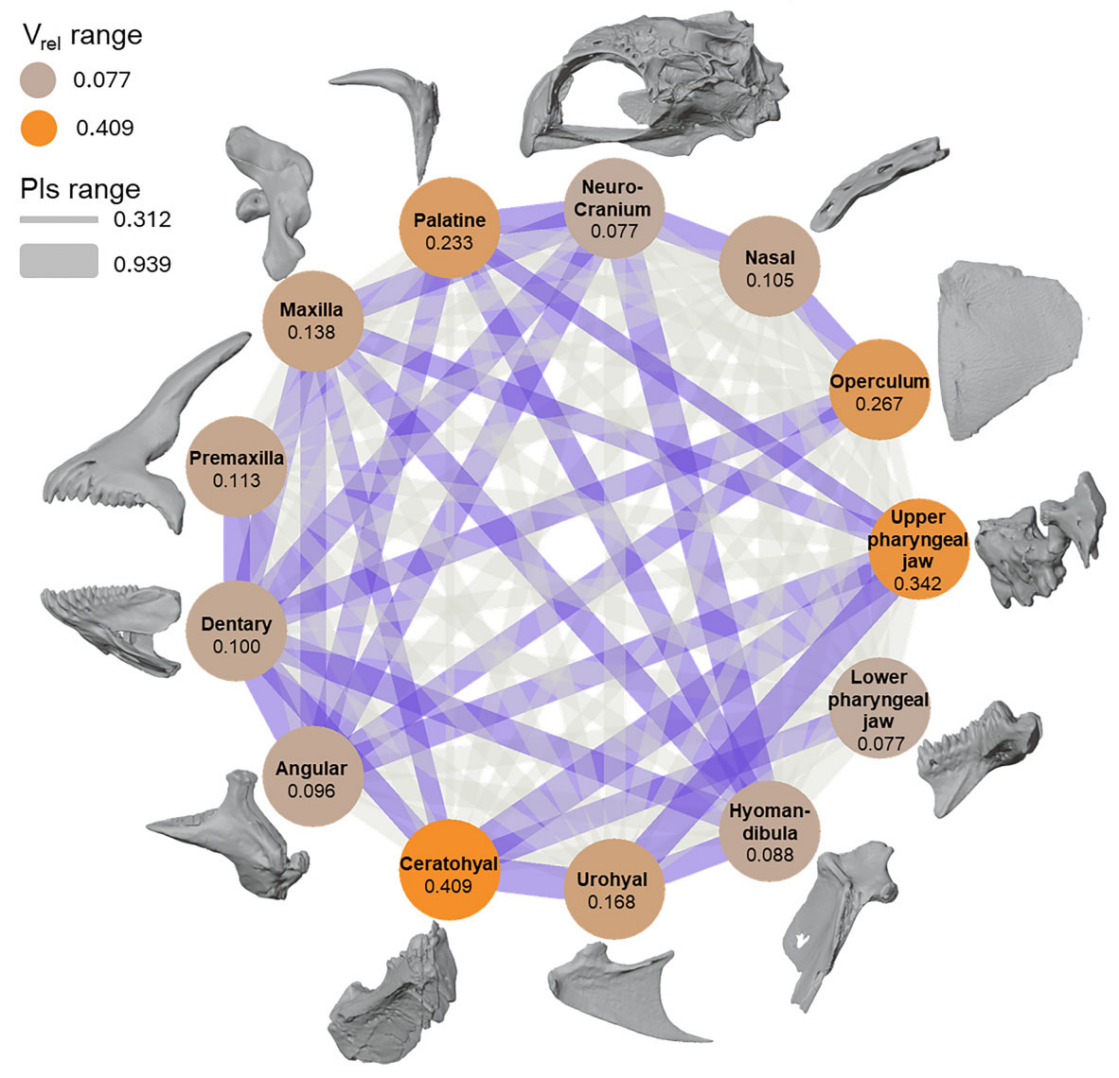
Network plot of the pls scores generated from the integration.test function in package geomorph. Line weight is indicative of pls scores with wider lines representing larger pls values. Statistically significant between-bone integration is indicated by the darker lines. Vertices represent within bone integration as calculated by the relative eigenvector index (V_rel_).

The ceratohyal and the upper pharyngeal jaw respectively had the highest degree of integration of the cranial bones analyzed as indicated by V_rel_. Integration between bones was calculated using rPLS values and revealed several statistically significant, between-bone associations (Fig. 5). The largest between-bone integration value (rPLS = 0.939) was recovered for the ceratohyal and the upper pharyngeal jaw, followed by the neurocranium and the hyomandibula (rpls = 0.873, z = 3.761), and then the angular and dentary (rpls = 0.864, z = 3.605).

### Morphological disparity

Patterns of morphological disparity between the five modules of the Developmental 2 hypothesis and across cranial bones were generally similar with a few exceptions (Tables S2 and S3). We find that the operculum module exhibits the most morphological disparity among modules tested. Among other modules the mandible (dentary and articular/angular) exhibits the second most morphological disparity following the operculum. Disparity values among the other modules were similar (Table S2). Among individual bones, we find more variation in patterns of morphological disparity (Table S3). Here, we find that the premaxilla, neurocranium, ceratohyal, and hyomandibula exhibit low levels of morphological disparity, while the opercle, maxilla, dentary, articular/angular, and nasals exhibit higher levels of morphological disparity. Interestingly, we find larger differences in morphological disparity between the upper and lower pharyngeal jaws, where the lower pharyngeal jaw exhibits less disparity than the upper pharyngeal jaw.

## Discussion

### The oral and pharyngeal jaws are decoupled in the lunar wrasse

Pharyngognathy was originally hypothesized to function as a modularizing force across the fish skull that decoupled the oral and pharyngeal jaws from each other, allowing for functional specialization and differentiation between the jaw sets (Kaufman and Liem 1982). However, recent evolutionary studies have cast doubt on the classic decoupling hypothesis, instead recovering strong patterns of evolutionary integration between oral and pharyngeal jaws. Here, we evaluated modularity patterns at the population level in the lunar wrasse and surprisingly, we recover strong support for the decoupling hypothesis. In our analysis, we find the strongest support for a modularity hypothesis that divided the skull based on a developmental origin scheme, comprised of modules containing the dorsal and ventral mandibular arch derivates, the neurocranium, and the hyoid arch derivatives. Furthermore, we find that integration between the two jaw systems is not supported based on significance testing. These results suggest that patterns of trait covariation introduced during development are maintained to some degree and structure the modularity of the lunar wrasse skull at the population level.

These results contradict many studies of pharyngognathy at the macroevolutionary level, which typically recover strong patterns of integration between the jaw systems. However, our findings are similar to other studies, which examined patterns of integration at the population level in cichlids and three species of pharyngognathous pupfishes (Hulsey et al. 2006; Chan et al. 2024). In cichlids, the oral and pharyngeal jaws were found to be decoupled despite their strong patterns of integration at the macroevolutionary level (Hulsey et al. 2006). In the study by Chan et al. (2024), the authors found strong support for a functional hypothesis that separated the oral and pharyngeal jaws within the populations of three pupfishes from the Bahamas. The authors hypothesized that the functional interactions within the two jaws systems, coupled with tissue-tissue interactions produced strong localized patterns of covariation within the skull that separated the oral and pharyngeal jaws into distinct modules.

Early during the development of the pharynx in vertebrates, the patterning of the pharyngeal arches is determined by complex interactions between different regions of the neural tube, migrating neural crest cells, *hox* gene expression and environmental cues from the endoderm. These arches eventually form distinct structures with semi-distinct cell fates. At this stage, the first and posterior pharyngeal arches are already incredibly modular with respect to each other (Graham 2003). However, developmental patterns of modularity can be quickly overwritten by tissue–tissue interactions (Hallgrímsson et al. 2009; Conith et al. 2021). The derivatives of these arches form the foundation of not only the skeletal elements associated with each arch, but also the muscular and nervous structures that form to support these skeletal elements. These sets of muscles have been shown to function fairly independently (Liem 1973; Westneat 2003; Datovo and Vari 2013). Additionally, recent studies have shown that soft-tissue interactions among bones can directly influence not only the shapes of bones but also the patterns of covariation between bones (Conith et al. 2019, 2021; Lofeu et al. 2024). In our study, a developmental partitioning of the skulls bones was the strongest supported model of modularity. However, the model with the second-best effect size was a functional hypothesis that separated the oral and pharyngeal jaws into distinct modules suggesting that the localized functional interactions within the two jaw systems may also play a role in structuring and reinforcing modularity between the oral and pharyngeal jaws. It is more likely that the skull of the lunar wrasse and perhaps many other pharyngognathous fishes represent a complex and dynamic palimpsest of developmental and functional interactions that allow the skull to perform multiple simultaneous functions in life while allowing it to evolve in a coordinated fashion across generations.

Our findings complicate our understanding of the relationship between variational and macroevolutionary patterns of modularity. One expectation might be that patterns of modularity observed at the population level should be reflected at the macroevolutionary level, since evolution occurs at the population level. However, studies have shown that the differences in scale between population and between-species comparisons can result in different patterns of trait covariation between these scales (Badyaev et al. 2005; Conith et al. 2021; Evans et al. 2023). Additionally, different processes (like plasticity) are at play at the population level that may depart from otherwise standard patterns of genetic inheritance and subsequent co-evolution (Zelditch and Carmichael 1989; Zelditch et al. 1992; Zelditch and Goswami 2021). It is possible that the oral and pharyngeal jaws develop and function in a modular fashion at the population level but co-evolve together as a result of a shared gene interaction network that forms the dentition on both sets of jaws (Fraser et al. 2009).

### Developmental origin as a constraint on phenotypic variation

Variation is considered to be the raw material for selection to act upon within a population (Schluter 1996, 2000). Genes or traits that exhibit more variation are more evolvable and available for selection to act upon them and thus, are more likely to evolve in response to selective pressures. At the phenotypic level, the developmental program is largely responsible for production of trait variation, and during development, tissues and progenitor cell populations can differ in the amount of phenotypic variation that they exhibit in the resulting adult phenotype (Yampolsky and Stoltzfus 2001; Evanset al. 2017a). At the macroevolutionary level, these traits that exhibit more variation may also exhibit higher rates of evolution (Goswami et al. 2023). Recent studies have suggested that the *hox-*derived pharyngeal jaws may constrain the evolution of the trophic apparatus due to the functional importance and highly conserved nature of *hox* genes during development and the important role that they play in determining early segmentation patterns across the body (Graham 2003; Fraser et al. 2009; Larouche et al. 2023; Nicholas and López-Fernández 2024). Here, we found differences in morphological disparity between several of our developmental modules, most notably between the mandible and the pharyngeal jaws with the mandible exhibiting more morphological disparity than the pharyngeal jaws. These results suggest that the pharyngeal jaws may be under stronger constraints and thus exhibit less variation within the lunar wrasse population potentially due to their location in branchial arches, which form in a region that is under strong influence from *hox* genes during development.

## Conclusion

Patterns of integration and modularity at the population level may be the reflection of both developmental and functional relationships that interact with environmental stimuli to shape adult phenotypes. As a result, these variational patterns of modularity can yield important insights into how organisms compartmentalize their traits in their respective environments. Pharyngognathy represents an interesting case of functional specialization, where the developmentally distinct oral and pharyngeal jaws perform specific roles in prey capture and prey processing. Macroevolutionary studies have frequently recovered strong evidence for the co-evolution of these structures over time despite their functional distinctions. In our study of the variational modularity of the lunar wrasse however, we find strong evidence for modularity between the oral and pharyngeal jaw structures following a developmental hypothesis that separates the first and second pharyngeal arch derivatives. The separation of these arches is highly conserved across vertebrates and is maintained through a series of complex developmental cues from the neural tube, migrating neural crest cells, *hox* gene expression, and the endoderm in developing embryos. Additionally, the soft tissue interactions that govern the function of these structures likely imparts other more localized patterns of covariation that can further modularize these structures. Our findings suggest that the factors that influence patterns of trait covariation at the population level may not directly translate to the macroevolutionary level because of developmental and environmental interactions that can shape the phenotypes of individuals at the population level that differ from patterns of co-inheritance at the macroevolutionary level.

## Author contributions

April Hugi: Investigation, Software, Formal analysis, Data Curation, Writing – Original Draft, Writing – Review & Editing, Visualization, Project administration. Howan Chan: Software. Andrea Rummel: Investigation. Hiroyuki Motomura: Resources, Funding acquisition. Yuna Dewa: Investigation, Midori Matsuoka: Investigation, Masayuki C. Sato: Investigation, Olivier Larouche: Software. Kory M. Evans: Conceptualization, Investigation, Resources, Writing – Original Draft, Writing – Review & Editing, Funding acquisition.

## Supplementary Material

icaf099_Supplemental_File

## Data Availability

The data underlying this article will be shared on reasonable request to the corresponding author.

## Notes

“From the symposium “Organismal Systems Biology” presented at the annual meeting of the Society for Integrative and Comparative Biology, January 3-7th, 2025”.
