# Supplementary material for: Developmental and Functional Interactions Structure Patterns of Variational Modularity in the Lunar Wrasse Skull
Source: Integr Comp Biol. 2025 Jun 20;65(3):560–71. doi: 10.1093/icb/icaf099 (PMC12464816; doi:10.1093/icb/icaf099)
Supplement: icaf099_Supplemental_File [file icaf099_supplemental_file.docx]

**Supplementary Tables**

**Table S1.** Descriptions of homologous anatomical landmarks and curves for sliding semi-landmarks adapted from LaRouche et al. 2023.

| **Homologous anatomical landmarks** | |
| --- | --- |
| **Landmark** | **Definition** |
| 1 | Most distal point of first tooth on premaxilla |
| 2 | Base of first tooth |
| 3 | Most distal point of ascending process of premaxilla |
| 4 | Proximal vertex between ascending and descending processes of premaxilla |
| 5 | Most distal point of descending process of premaxilla |
| 6 | Most distal point of first tooth on dentary |
| 7 | Posterior-most point on dentary flange |
| 8 | Ventral-most point of mental symphysis on dentary |
| 9 | Antero-distal-most point of articular/angular |
| 10 | Distal-most point of coronoid process |
| 11 | Ventral, posterior-most point of dentary |
| 12 | Center of jaw joint on quadrate |
| 13 | Posterior-most point on retroarticular |
| 14 | Most anterior point of vomer |
| 15 | Left, distal-most point on anterior of vomer (used to approximate width of vomer) |
| 16 | Proximal lachrymal-lateral ethmoid margin |
| 17 | Proximal lateral ethmoid-parasphenoid margin |
| 18 | Dorsal-most point of lachrymal |
| 19 | Proximal-most point of lateral ethmoid frontal margin |
| 20 | Origin of supraoccipital |
| 21 | Posterior frontal sub-orbital margin |
| 22 | Pterosphenoid-parasphenoid margin |
| 23 | Mid-point parasphenoid-basioccipital margin |
| 24 | Prootic foramen |
| 25 | Frontal parietal margin |
| 26 | Posterior-most point of supraoccipital |
| 27 | Ventral epiotic-post temporal margin |
| 28 | Distal-most dorsal point of basioccipital |
| 29 | Pterotic-post temporal margin |
| 30 | Distal-most ventral point of basioccipital |
| 31 | Anterior most point of lower pharyngeal jaw plate |
| 32 | Descending process of lower pharyngeal jaw plate |
| 33 | Lateral-central most point of pharyngeal tooth plate |
| 34 | Ventral base of descending process of pharyngeal tooth plate |
| 35 | Lateral wing of pharyngeal tooth plate |
| 36 | Posterior center-most point of pharyngeal tooth plate |
| 87 | Distal,dorsal anterior margin of maxilla |
| 88 | Distal,dorsal posterior margin of maxilla |
| 89 | Distal, ventral anterior margin of maxilla |
| 90 | Distal, ventral posterior margin of maxilla |
| 91 | Proximal,dorsal anterior margin of maxilla |
| 92 | Proximal,dorsal posterior margin of maxilla |
| 93 | Anterior-most point of the proximal descending process of maxilla |
| 94 | Anterior-most point of contact on the distal face of the hyomandibula between hyomandibula and sphenotic |
| 95 | Posterior-most point of contact on the distal face of the hyomandibula between the hyomandibula and the pterotic |
| 96 | Distal-most point of lateral projection of hyomandibula |
| 97 | Anterior-ventral most point of hyomandibula |
| 98 | Ventral-most point of hyomandibula |
| 99 | Anterior, proximal-most point of nasal |
| 100 | Anterior, distal-most point of nasal |
| 101 | Posterior, proximal-most point of nasal |
| 102 | Posterior, distal-most point of nasal |
| 150 | Dorsal, anterior-most point of urohyal |
| 151 | Ventral anterior-most point of urohyal |
| 152 | Dorsal, posterior-most point of urohyal |
| 153 | Ventral, posterior-most point of urohyal |
| 154 | Anterior-most point of ceratohyal |
| 155 | Interior-ridge of ceratohyal |
| 156 | Dorsal ceratohyal-epihyal margin |
| 157 | Posterior-most point of epihyal |
| 158 | Ventral ceratohyal-epihyal margin |
| 179 | left outside angle of preoperculum |
| 180 | left anterior tip of preoperculum |
| 181 | left interopercle-opercle joint |
| 182 | left joint between preoperculum and neurocranium |
| 183 | left joint between hyomandibula and operculum |
| 184 | left anterior tip of palatine |
| 185 | left dorsal point on curve of palatine |
| 186 | left ventral tip of palatine at pterygoid joint |
| 187 | left dorsal tip of palatine at pterygoid joint |
| 188 | anterior tip of hypohyal |
| 189 | posterior tip of hypohyal |
| 190 | joint between hypohyal and ceratohyal |
| 191 | left dorsal tip of interhyal |
| 192 | left side of joint between upper pharyngeal jaws |
| 193 | left dorsal point of upper pharyngeal jaws |
| 194 | left ventral point of upper pharyngeal jaws |
| 195 | left lateral point on upper pharyngeal jaws |
| 196 | left upper pharyngeal jaw joint |
| 197 | left uppermost tip of upper pharyngeal jaw |
| **Homologous curves for the positioning of sliding semi-landmarks** | |
| **Landmark** | **Definition** |
| 37-47 | Curve 1: Ascending process of premaxilla, between landmarks 2 and 3 |
| 48-51 | Curve 2: Ventral margin of dentary, between landmarks 8 and 11 |
| 52-62 | Curve 3: Ventral margin of parasphenoid, between landmarks 14 and 23 |
| 63-77 | Curve 4: Supraoccipital crest, between landmarks 20 and 26 |
| 78-86 | Curve 5: Orbital margin, between landmarks 18 and 21 |
| 103-106 | Curve 6: Angular ascending process, between landmarks 10 and 12 |
| 107-110 | Curve 7: Angular lateral process, between landmarks 9 and 12 |
| 111-115 | Curve 8: Lateral arm of lower pharyngeal tooth plate, between landmarks 34 and 35 |
| 116-120 | Curve9: Descending process of lower pharyngeal tooth plate, between landmarks 34 and 32 |
| 121-126 | Curve 10: Descending arm of premaxilla, between landmarks 4 and 5 |
| 127-131 | Curve 11: Lateral arm of premaxilla, between landmarks 2 and 5 |
| 132-135 | Curve 12: Anterior face of hyomandibula, between landmarks 94 and 97 |
| 136-139 | Curve 13: Dorsal surface of hyomandibula, between landmarks 94 and 95 |
| 140-143 | Curve 14: Posterior face of hyomandibula, between landmarks 96 and 98 |
| 144-146 | Curve 15: Nasal interior surface, between landmarks 99 and 101 |
| 147-149 | Curve 16: Nasal outer edge, between landmarks 100 and 102 |
| 159-163 | Curve 17: Urohyal dorsal edge, between landmarks 150 and 152 |
| 164-168 | Curve 18: Urohyal ventral edge, between landmarks 151 and 153 |
| 169-173 | Curve 19: Ceratohyal dorsal ridge, between landmarks 154 and 156 |
| 174-178 | Curve 20: Ceratohyal ventral ridge, between landmarks 154 and 158 |

**Table S2.** Morphological disparity of the hypothesized lunar wrasse skull modules which received the strongest support in this study.

| **Developmental 2 Hypothesis Modules** | **Morphological Disparity** |
| --- | --- |
| Premaxilla, Maxilla | 1.413419e-07 |
| Dentary, Articular | 8.692633e-07 |
| Ceratohyal, Urohyal, Hyomandibula, Pharyngeal jaws | 2.776813e-07 |
| Operculum | 4.998264e-06 |
| Neurocranium, nasal | 2.551052e-07 |

**Table S3.** Morphological disparity of individual lunar wrasse skull bones.

| **Bone** | **Morphological Disparity** |
| --- | --- |
| Premaxilla | 9.072634e-08 |
| Maxilla | 1.981584e-07 |
| Dentary | 2.264668e-07 |
| Articular | 1.219437e-07 |
| Nasal | 1.585268e-07 |
| Neurocranium | 2.991071e-08 |
| Hyomandibula | 9.325103e-08 |
| Ceratohyal | 8.343513e-08 |
| Urohyal | 1.132334e-07 |
| Operculum | 4.998264e-06 |
| Upper pharyngeal jaws | 2.642113e-07 |
| Lower pharyngeal jaws | 9.907922e-08 |
